# Lipase-Catalyzed Acidolysis of Egg-Yolk Phosphatidylcholine with Citronellic Acid. New Insight into Synthesis of Isoprenoid-Phospholipids

**DOI:** 10.3390/molecules23020314

**Published:** 2018-02-02

**Authors:** Magdalena Rychlicka, Natalia Niezgoda, Anna Gliszczyńska

**Affiliations:** Department of Chemistry, Wroclaw University of Environmental and Life Sciences, Norwida 25, 50–375 Wrocław, Poland; rychlicka.magda92@gmail.com (M.R.); natalianiezgoda86@gmail.com (N.N.)

**Keywords:** acidolysis, egg-yolk phosphatidylcholine, citronellic acid, isoprenoids, lipase, anticancer activity

## Abstract

The development of a biotechnological method for the production of new biologically active phosphatidylcholine containing monoterpene citronellic acid (CA) was the aim of this work. Incorporation of citronellic acid (CA) into egg-yolk phosphatidylcholine (PC) in the lipase-catalyzed acidolysis process was studied. Isoprenoid acid CA was used as an acyl donor and five commercially available immobilized lipases were examined as biocatalysts. The effects of organic solvent, enzyme load, reaction time and molar ratio of substrates on the incorporation of citronellic acid (CA) into the phospholipids were evaluated. Modified phospholipid fraction enriched with CA in the *sn*-1 position (39% of incorporation) was obtained in high 33% yield using Novozym 435 as biocatalyst. In this study a biotechnological method for production of new phospholipid biopreparation enriched with citronellic acid, which can play an important role as a nutraceutical, was applied.

## 1. Introduction

Isoprenoids are a wide group of natural compounds occurring, both in vegetal and animal species, where they play important biological functions such as pheromones, phytoalexins and antifeedants [1,2]. Over the last few decades, the broad spectrum of therapeutic properties of isoprenoids useful for treatment of human diseases has been the subject of many papers. Isoprenoids are well known as immunomodulatory, anti-inflammatory, antitumor or antimicrobial agents [3,4,5,6]. Among the mentioned properties, chemopreventic and anticancer activities of isoprenoid compounds should be especially highlighted. A number of dietary terpenes have antitumor activity. They exhibit not only the ability to prevent the formation or progression of cancer but also the ability to regress existing malignant tumors [7]. Therefore, drugs derived from isoprenoid compounds have contributed significantly to human disease therapy and for that reason this group of natural products arouses great interest. The most renowned terpene-based drug for anticancer therapy is Taxol^®^ which is used in the treatment of ovarian, lung and breast cancer [8]. Recent studies have shown that monoterpenes exert antitumor activities and indicate that these compounds could be a new class of cancer chemopreventive agents [7,9]. Perillyl alcohol, which is in the second phase of clinical trials exhibits significant ability to induce apoptosis of prostate cancer cells either in in vitro and in in vivo tests [10]. Another well-established chemopreventive and therapeutic agent against many cancer cell lines being under the clinical trial (phase I and phase II) is limonene [7]. Acyclic isoprenoids such as geraniol, farnezol or geranylogeraniol exhibit antiproliferative activity towards selected tumor cell lines as well [11,12,13]. Their potency to inhibit tumor growth has been documented to be significantly greater than activity of cyclic isoprenoids [7]. These compounds inhibited growth of cells with impaired proliferation but did not affect the growth of the healthy ones, what distinguish them from commercially available cytostatic such as cisplatin.

Slightly less known in the literature, but having also promising biological properties is structural analogue of geraniol-citronellic acid (CA) (3,7-dimethyl-6-octanoic acid). This aliphatic, monoterpene acid has been found along with geraniol in essential oils of lemongrass and citrus fruits [14]. Large amount of CA was also detected in essential oils of two *Pelargonium* species: *P. papilionaceum* and *P. vilifolium* [15,16]. Citronellic acid is widely used in cosmetic industry not only as a fragrance ingredient of various products but also as an agent, which exhibits the broad antimicrobial spectrum [17].

Monoterpenes exhibit very high degree of oral bioavailability in mammals [18]. Unfortunately, these compounds have problems, due to short blood half-life resulted in their lower activity in the biological systems [19]. However, terpenes activity could be enhanced by attaching them to phospholipid molecules. Previous studies have indicated that terpene-phospholipids exhibit significantly higher antiproliferative activity than free terpenes [20,21,22,23]. It was proven that phosphatidylcholine covalently bonded with citronellic acid (CA) exhibits from 4 to 9-fold higher antiproliferative activity towards human leukaemia (MV4-11), lung (A-549), breast (MCF-7), liver (HepG2) and colon (LoVo and LoVo/DX) cancer cell lines in comparison with free form of this terpene acid. The highest activity towards MV4-11, A-549, LoVo and LoVo/DX lines was especially observed for hetero-substituted phospholipid containing citronellic acid in the *sn*-1 position and palmitic acid in the *sn*-2 position of PC, which was obtained by the chemical method. These results confirm documented in the literature data that phospholipids (PLs) enhance activity of therapeutic agents attached to them by changing their solubility and stability [24]. Moreover, due to non-toxicity of PLs, their nutritional value, significant compatibility to cell membranes and capacity to generate different supramolecular structures it is justified to combine the valuable activities of these two groups of compounds.

In the literature, there are only a few reports presenting the methods of production of isoprenoid-phospholipids by chemical synthesis [22,23,25] and only two concerning the enzymatic synthesis of this type of biomolecules [20,21]. Yamamoto and coworkers studied enzymatic incorporation of terpene alcohols into the hydrophilic region of phospholipids in the transphosphatidylation reaction catalyzed by phospholipase D and they observed increased antitumor activity of terpene-phospholipid derivatives compared with free monoterpene alcohols [20,21]. These findings undoubtedly have expanded terpenes applicability, however, the strategy of modification based on the replacement of polar region of phosphatidylcholine (PC) with isoprenoid compounds leads to loss of valuable choline. Therefore, from the beneficial health point of view more appropriate approach is the introduction of therapeutic compound into the hydrophobic region of phospholipid.

Driven by the encouraging results achieved during the biological evaluation of the anticancer activity of synthesized 1-citroneloyl-2-palmitoyl-sn-glicero-3′-phosphatidylcholine, in this study we attempted to introduce the natural citronellic acid into the structure of natural egg-yolk phosphatidylcholine (PC) and developed new pharmaceutical bioproduct which can improve human health. In our project we elaborated the incorporation of CA into PC using regioselective lipases to replace the fatty acids occur at position *sn*-1 of natural PC with citronellic acid.

The presented paper makes a substantial contribution to the research area concerning the production of structured PLs containing biologically active compounds other than fatty acids using the substrates of natural origin. The objective of this study was also to demonstrate that the natural citronellic acid could be used as an acyl donor, which is acceptable for lipases in the enzymatic acidolysis of phospholipids. In order to optimize these process parameters such as: organic solvent, lipases, enzyme load, substrate molar ratio and reaction time were studied.

## 2. Results and Discussion

In the production of structured phospholipids with the defined fatty acids profile, having biological applications, the enzyme-catalyzed reactions have the decisive advantage over chemical reactions. Our studies were aimed at the development of the enzymatic synthesis of new phospholipids enriched with citronellic acid (CA) since the antiproliferative activity towards selected cancer cell lines for these conjugates have been confirmed [22]. As a method of modification, direct one-step, direct lipase-catalyzed acidolysis of egg-yolk phosphatidylcholine with CA was selected. The enzyme reaction was designed to amplify the therapeutic potential of natural egg-yolk phosphatidylcholine resulting from the presence of unsaturated fatty acids and choline in *sn*-2 and *sn*-3 positions respectively. This type of modification strategy was based on the incorporation of biologically active citronellic acid into the *sn*-1 position of non-polar region of native PC where predominantly saturated fatty acids occur. Citronellic acid has been used as an acyl donor in the conducted enzyme reactions. Type of modifications presented in this paper and new PLs biomolecule with isoprenoid acid in the *sn*-1 position obtained by this method has not yet been described in the literature. In our experiments, attention was paid to obtain the highest degree of incorporation of CA into the phosphatidylcholine structure.

### 2.1. Screening of Lipases

**Figure 1:**
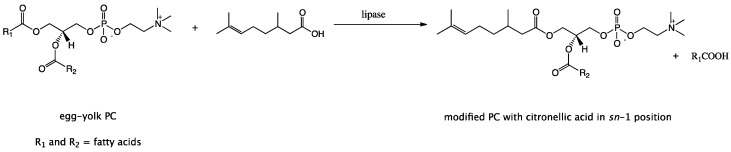
Lipase-catalyzed acidolysis of egg-yolk phosphatidylcholine with citronellic acid.

Immobilized lipases have a wide range of applications in lipid modification studies and they offer advantages of higher catalytic activity and stability in organic solvent medium over other enzymes [26]. Therefore, five commercially available immobilized lipases from: *Candida antarctica* (CALA, CALB, Novozym 435), *Rhizomucor miehei* (Lipozyme^®^) and *Thermomyces lanuginosus* (Lipozyme TL IM) were tested in terms of their ability to catalyze acidolysis reactions between egg-yolk PC and citronellic acid Figure 1.

These biocatalysts were selected based on the literature data indicating that mentioned lipases effectively catalyze the structural modifications of natural triacylglycerols and natural or synthetic phospholipids [27,28,29]. Reaction conditions were also established after the analysis of literature data, suggesting that in order to enrich the PC with natural compounds other than fatty acids, it is necessary to use much higher molar excess of acyl donors and toluene as the reaction medium [29,30]. The enzymatic acidolysis reaction was then carried out at 30 °C using a 1:30 PC/CA molar ratio, 20% enzyme dosage and toluene as solvent. Keeping in mind that it is important to reduce the total costs of process while choosing an enzyme for industrial applications in our experiments we decided to apply enzymes at the same weight ratio although they exhibit different activities (according to suppliers).

The lipases used in studies are known as 1,3-regioselective biocatalysts in this type of reactions. Using them for modifications of non-polar part of egg-yolk PC we could then obtain the theoretical maximum of incorporation of citronellic acid into PC on the level of 50%.

**Figure 2:**
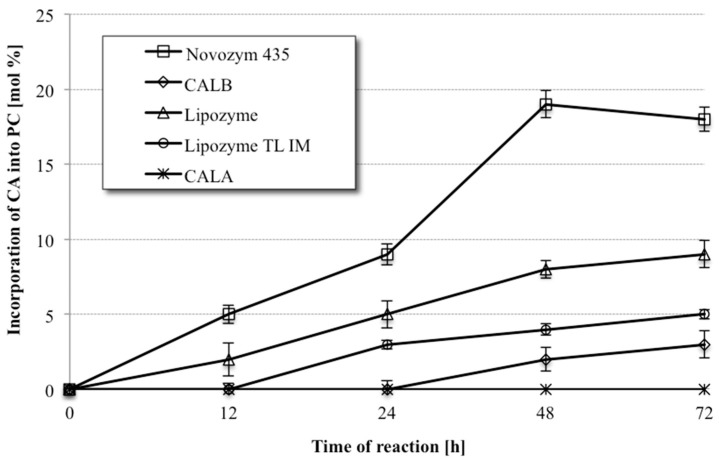
Enzyme screening for acidolysis of egg yolk phosphatidylcholine (PC) with citronellic acid (CA) (reaction conditions: 1:30 PC/CA molar ratio, enzyme load 20% (*w*/*w*), toluene 2 mL, temperature 30 °C).

The progress of the reactions was monitored by collecting samples of the product mixtures after allowing the reactions to proceed for chosen length of time (12, 24, 48 and 72 h). The products mixtures were subsequently subjected to the solid phase extraction (SPE) to aid separation of PLs from the acids. In the next step, the fatty acid composition in the PL fraction was analyzed by gas chromatography (GC), whereas the information about the proportion between the modified and native PC was provided by high-performance liquid chromatography (HPLC) analysis. In these initial studies the PL were not fractionated into individual PC and lysophosphatidylcholine (LPC), which is formed in this process due to partial hydrolysis of phosphatidylcholine during the acidolysis reaction. The time course of the incorporation of CA into PC by lipases is presented in Figure 2. It can be observed that only lipase B from *C. antarctica* immobilized on a macroporus acrylic resin (Novozym 435) was able to produce the desired PC with high incorporation level among all tested biocatalysts. This enzyme exhibits the highest activity in PC modification giving 19% incorporation of CA into phospholipid fraction after 48 h of reaction. Preparations of Lipozyme^®^, Lipozyme TL IM and CALB, showed significantly lower activity and the CA incorporation into phospholipid fraction did not exceed 9%, whereas lipase A from *C. antarctica* was inactive in acidolysis process. Novozym 435 was therefore used as the biocatalyst on the synthesis of phospholipid biopreparation enriched with citronellic acid for the following experiments.

### 2.2. Effect of Organic Solvent

In general, a type of solvent can strongly influence on the enzymatic reaction in different ways affecting water availability for enzyme and denaturation of enzyme protein [31]. Moreover, polarity of solvents could also affect the solubility of substrates [32]. Well-selected organic solvent for acidolysis reaction should limit the availability of water for lipase inhibiting the competitive hydrolysis reaction and therefore hydrophobic solvents are considered to be a better medium for esterification reactions than hydrophilic ones [26]. Among appropriate solvents that can be used in the reaction of acidolysis the highest incorporation of fatty acids into PC or other biologically active molecules like *n*-3 fatty acids or lipoic acid was observed mostly when the reaction medium was toluene or heptane, strongly hydrophobic solvents [29,30,33,34]. The effect of these solvents on the Novozym 435-catalyzed acidolysis of egg-yolk PC with CA was investigated.

**Figure 3:**
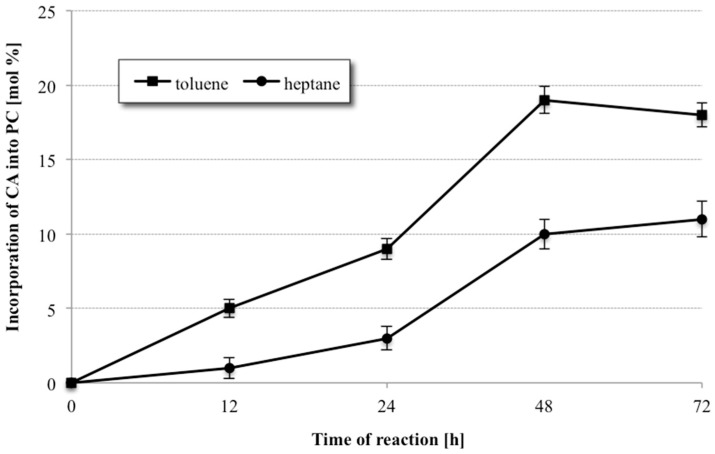
Effect of different organic solvent on the incorporation of CA into egg-yolk PC in acidolysis reaction (reaction conditions: 1:30 PC/CA molar ratio, enzyme load 20% (*w*/*w*) Novozym 435, solvent toluene/heptane 2 mL, temperature 30 °C).

As shown in Figure 3, the reaction was very medium dependent. When the reaction was carried out in heptane, a relatively low degree of CA incorporation only 11% after 72 h into phospholipid fraction was observed. A significantly higher degree of incorporation of citronellic acid was obtained for the reaction carried out under the same conditions in toluene. Using toluene as the reaction medium resulted in increase of terpene acid incorporation from 11 to 19% in shorter time 48 h. When the reaction was continued for another 24 h, a decrease in the degree of incorporation of CA into PC was observed, probably as the results of partial hydrolysis of previously formed structured phosphatidylcholine. Due to the obtained results, toluene was chosen as the reaction medium in the further experiments.

### 2.3. Substrate Molar Ratio

**Figure 4:**
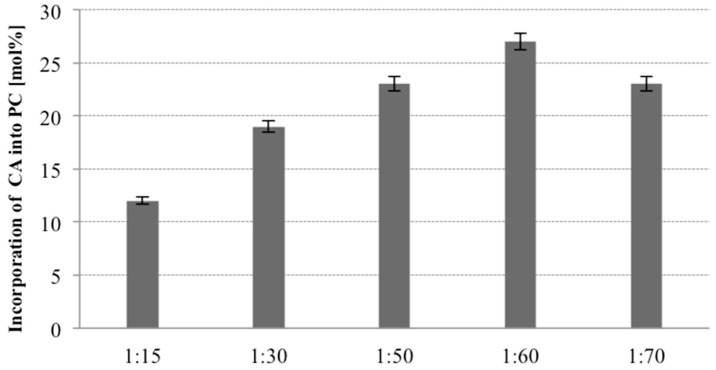
Effect of substrate molar ratio on the incorporation of CA into egg-yolk PC in acidolysis reaction (reaction conditions: PC 30 mg, 1:15/1:30/1:50/1:60/1:70 PC/CA molar ratio, toluene 2 mL, temperature 30 °C, enzyme load 20% (*w*/*w*) Novozym 435, time 48 h).

The PL composition of the product in the enzymatic acidolysis reaction depends also on the substrate ratio (mol PL/mol acyl donor). The ratio of PC to citronellic acid was varied from 1:15, 1:30, 1:50, 1:60 and 1:70 (mol PC/mol citronellic acid) and the influence of this parameter on the degree of terpene acid incorporation into PC was evaluated. For these studies Novozym 435 and optimized medium were used. In all studied variants, other conditions of 30 °C temperature, 48 h of reaction time and 20% (*w/w*) of lipase were stable. As shown in Figure 4, increasing the molar ratio of CA to PC led to a gradual increase of the incorporation level of terpene acid from 12% (1:15) to 27% (1:60). It was observed that a high concentration of terpene acid was necessary for obtaining greater incorporation. These results are in accordance with the literature data indicating that high substrate molar ratio shifts the reaction equilibrium to the product side and improve the incorporation [35]. However, in our case no further increase of incorporation was detected when the molar ratio was 1:70 (PC to CA). It means that beyond concentration 1:60 the activity of enzyme is reduced and the decrease in the level of incorporation is observed. High substrate molar ratio increases also the cost of the process and could result in difficulties of separation of the products, but selected in our experiments substrate molar ratio of 1:60 (PC/CA) was justified by the high degree of incorporation and did not create problems with separation therefor this parameter was chosen for the following study.

### 2.4. Enzyme Dosage

In the next step of optimization of enzymatic production of phospholipid preparation enriched with citronellic acid we focused on the effect of enzyme load on the process of acidolysis of PC. The effect of the enzyme dosage on the level of incorporation was evaluated by varying amount of Novozym 435 from 20 to 40 weight (wt) % (based on substrates). It has been reported that high dosage of enzyme significantly influences the incorporation degree [36,37].

**Figure 5:**
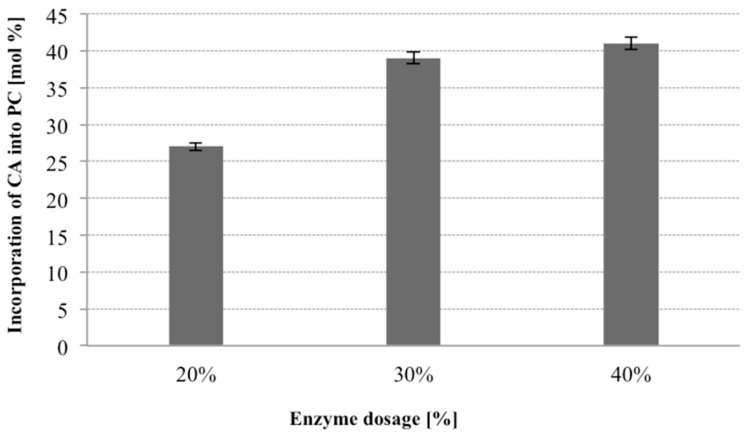
Effect of enzyme load on the incorporation of CA into egg-yolk PC in acidolysis reaction (reaction conditions: PC 30 mg, 1:60 PC/CA molar ratio, toluene 2 mL, temperature 30 °C, enzyme load 20/30/40% (*w*/*w*) Novozym 435, time 48 h).

The effect of different enzyme loadings can be seen in Figure 5, which is a graphical presentation of how the CA incorporation levels into the total PL were affected after 48 h of the reaction. When relatively low dosages of the lipase were employed, relatively minor changes seemed to take place in the fatty acid composition. As the enzyme load was increased from 20% to 30% there was an obvious increase of the incorporation of CA into phospholipids from 27% to 39%. The highest level of incorporation was observed by employing 40% dosage of lipases (41% of incorporation), however in this case CA incorporation increased marginally. The content of terpene acid in modified phospholipid fraction was not significantly different irrespective of 30% or 40% enzyme used. Taking into account the economy of the process we decided to use the amount of 30% of the lipase in the further studies.

### 2.5. Reaction Temperature

The time course of enzymatic reactions is strongly correlated with the temperature in which the process is performed and could impact on it in several ways. Generally, this parameter increases the solubility of the substrates what usually translates into an increase in the degree of incorporation of tested acyl donors as well as enzyme activity [36]. On the other hand, each biocatalyst possesses strictly determined temperatures that are optimum and too high a temperature could cause irreversible denaturation of tested enzyme. Moreover, high temperature enhances side reactions such as acyl migration and hydrolysis [38]. Therefore, the applied temperature should be always consistent with the optimum for studied enzyme. According to the manufacturer (Sigma Aldrich, St. Louis, MO, USA), the optimal temperature for activity of Novozym 435 is between 30 and 60 °C. Taking this into account as well as the fact that the isoprenoid compounds are volatile, the optimization of this reaction parameter was carried out in two temperature variants 30 and 50 °C. The incorporation of citronellic acid into the phospholipid was observed at both tested trials. The enzyme activity was observed to drop sharply at 50 °C. It was determined that the process of modification of egg-yolk PC was much more effective at 30 °C (39% of incorporation) than at 50 °C (11% of incorporation).

### 2.6. Identification of Acidolysis Products

**Figure 6:**
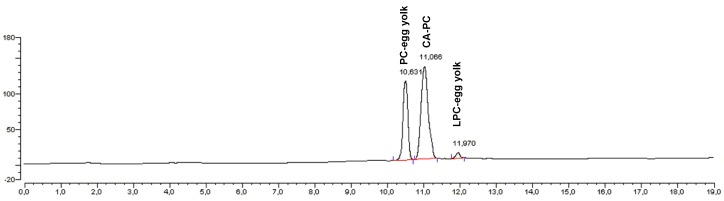
HPLC chromatogram of fraction of modified phospholipid.

The best method for identification of products of enzymatic modifications and the composition of modified phospholipid fractions in our experiments was HPLC equipped with a Corona charged aerosol detector (CAD). This method was chosen because we had the standards of products that can be formed during the acidolysis reaction: 1-citroneloyl-2-palmitoyl-*sn*-glicero-3′-phosphatidylcholine (1-CA-2-PA-PC), as an analogue of modified PC and 1-citroneloyl-2-hydroxy-*sn*-glicero-3′-phosphatidylcholine (1-CA-2-OH-LPC). Those products we have obtained previously by the chemical methods [22]. We used also as the standards native PC (PC-egg yolk) and product of its enzymatic hydrolysis (LPC-egg yolk). Below we present the HPLC chromatogram of products mixture (phospholipid fraction) (Figure 6) and fatty acids (wt %) profile of modified phospholipid fraction (Table 1) for the best variant of the reaction of acidolysis of PC with CA with optimized parameters: organic solvent (toluene), lipase (Novozym 435, enzyme load 30%), substrate molar ratio (PC:CA, 1:60) and reaction time (48 h).

Based on the HPLC chromatogram obtained from the sample derived after 48 h from reaction performed at optimized condition the extracted phospholipid fraction contained 68% of PC modified with CA (CA-PC), 29% of native PC (PC-egg yolk) and only 3% of product of its hydrolysis LPC-egg yolk. We did not observe modified lyso-PC which could be caused by high terpene acid concentration and hydrophobic solvent that were used in the experiments leading to inhibition of the formation of *sn*-glycerophosphocholine—the only substrate that can be used by enzyme in production of LPC with modified acyl in *sn*-1 position as it was reported earlier by Adlercreutz [39]. The CA-PC was obtained with high 33% yield. The degree of incorporation of citronellic acid was determined based on the changes in the composition of fatty acid profile in native PC and modified phospholipid fraction (Table 1). The increase of terpene acid CA was accompanied by a reduction in saturated fatty acids, which usually occupy the *sn*-1 position of egg-yolk PC.

## 3. Materials and Methods

### 3.1. Materials and Chemicals

Lohman Brown hens were a gift from the Tronina factory. Lipase B from *Candida antarctica* immobilized in a macroporus acrylic resin (synonym: Novozym 435 > 5000 U/g), lipase B from *Candida antarctica* (CALB > 1800 U/g) and lipase A from *Candida antarctica* (CALA > 500 U/g) both immobilized on resin Immobead 150 were purchased from Sigma-Aldrich (St. Louis, MO, USA). Immobilized lipase from *Rhizomucor miehei* (Lipozyme^®^ > 30 U/g) was provided by Fluka (Buchs, Switzerland) while immobilized lipase from *Thermomyces lanuginosus* (Lipozyme TL IM, 250 U/g) was supplied by Novozymes A/S (Bagsvaered, Denmark). Citronellic acid (CA) (3,7-dimethyl-6-octanoic acid, purity: 98%), a boron trifluoride methanol complex solution (13–15% BF_3_ × MeOH), sodium methylate and heptane were purchased from Sigma-Aldrich (St. Louis, MO, USA). The thin layer chromatography (TLC) pre-coated silica gel plates (Kieselgel 60 F_254_, 0.2 mm), the silica gel (Kieselgel 60, 230–400 mesh), solvents used in chromatography and HPLC grade solvents (Merck LiChrosolv^®^ Reag.) were purchased from Merck (Darmstadt, Germany).

### 3.2. Isolation of PC from Egg-Yolk

A crude phospholipid extract from egg yolk were isolated from hen (Lohman Brown) eggs on a semi-technical scale using the equipment from Wroclaw Technology Park. Eggs were obtained from the poultry farm “Ovopol” (Nowa Sól, Poland) and were dried in the drying chamber at inlet air temperature 185 ± 5 °C and an outlet air temperature 70 ± 2 °C. Obtained powder was subsequently extracted with ethanol in a tank equipped with a mechanical stir maintaining the dilution ratio of yolk to solvent at 1:4 (*m*/*v*). The process of suspension was carried out for 90 min. and then alcohol was removed by filtration. The residue was evaporated in vacuo (0.06 MPa at 50 °C). Next the crude extract of PLs (20 g) was subjected to silica gel column chromatography. A pure phosphatidylcholine (PC) fraction (*R_f_* 0.4) (11.6 g) was separated from extract by silica gel column chromatography using a mixture chloroform/methanol/water (65:25:4, *v*/*v*/*v*) as eluent. Purity of obtained PC was 99% according to HPLC and was also analyzed by TLC on silica gel-coated aluminium plates (chloroform/methanol/water (65:25:4, *v*/*v*/*v*)). Composition of fatty acids in isolated phosphatidylcholine was shown in Table 1.

### 3.3. The Lipase-Catalyzed Acidolysis of PC With Citronellic Acid

The reactions were carried out using of native egg yolk PC and were conducted in screw-capped vials. The egg-yolk PC (30 mg, 0.039 mmol) was mixed with citronellic acid (CA) at molar ratio 1:30 (PC/CA) in 2 mL of toluene and then 20% of lipase (by weight of substrates) was added. The reactions were carried out at 30 °C on a magnetic stirrer at 300 rpm and stopped at the selected time intervals by enzyme filtration on a G4 Shott funnel with Celite layer. The reactions were carried out using five different lipases, in N_2_ atmosphere. The effect of different organic solvent, molar ratio of substrates, lipase dosage and temperature was examined in another set of experiments for Novozym 435. All experiments were performed in triplicates.

### 3.4. Solid-Phase Extraction (SPE)—Separation of Phospholipid Fraction (PC/LPC)

Modified phospholipid fractions were purified and concentrated using SPE method according to the described procedure with slight modification [28]. A silica gel column (Discovery^®^ DSC-Si SPE, 52654—U 500 mg) was conditioned by successive washing with 10 mL of methanol, 10 mL of chloroform and 6 mL of chloroform/acetic acid (95:5, *v*/*v*). Evaporated reaction mixture (150 μL) was applied to SPE cartridge. The CA and fatty acids were eluted with 15 mL of chloroform/acetic acid (95:5, *v*/*v*). Then phospholipids were eluted with solvent mixtures of increasing polarity 10 mL of chloroform/propan-2-ol (1:1, *v*/*v*) and then with 35 mL of methanol/water (25:4, *v*/*v*). The phospholipid fraction was evaporated using a rotatory vacuum evaporator at 40 °C.

### 3.5. Analysis of Substrates and Products

Qualitative analysis of the reaction mixtures was made by TLC on silica gel-coated aluminum plates using as the developing system mixture of chloroform/methanol/water, 65:25:4, *v*/*v*/*v*). After elution, the plates were developed using the 0.05% primuline solution (acetone:water, 8:2, *v*/*v*) and spots were detected under an ultraviolet (UV) lamp (*λ* = 365 nm).

The reaction products were analyzed by HPLC using the method performed earlier for the synthetic pattern of phospholipids with citronellic acid [22].

Fatty acid (FA) profile of native PC and modified phospholipid fractions (PC/LPC) were analyzed by gas chromatography after their conversion to the corresponding methyl esters (FAME). Transesterification was conducted by heating under reflux (3 min) mixture of 10 mg of substrate with 3 mL of BF_3_ × MeOH complex solution. Next the mixture was cooled and products were extracted with 2 mL of hexane and the organic layer were washed with a saturated NaCl solution and dried over anhydrous MgSO_4_.

Methyl esters of fatty acid and isoprenoid acid were directly analyzed by gas chromatography (GC) on an Agilent 6890N with a flame ionization detector (FID) (Agilent Technologies, Santa Clara, CA, USA). The separation was achieved on DB-WAX column (30 m × 0.32 mm × 0.25 μm) manufactured by Agilent (Santa Clara, CA, USA). The initial oven temperature was 90 °C and was then increased to 250 °C at a rate of 5 °C/min and then held for 5 min. The total analysis time was 34 min. The injector temperature and the flame ionization detector were set at 250 °C. Hydrogen was used as a carrier gas with a constant flow 1.5 mL/min. The FAME were identified by comparing their retention times with those of a standard FAME mixture (Supelco 37 FAME Mix) purchased from Sigma Aldrich.

## 4. Conclusions

The present work is aimed at the production of 1-citroneloyl-phosphatidylcholine through the lipase-catalyzed reaction of egg-yolk PC with citronellic acid and optimizing the various reaction parameters that affect this reaction. The most effective enzyme able to catalyze this process of acidolysis was Novozym 435. Optimization of process parameters such as: organic solvent (toluene), lipase (enzyme load 30%), substrate molar ratio (PC:CA, 1:60) and reaction time (48 h) allowed to obtain product CA-PC in 33% yield in which the degree of citronellic acid incorporation into the PC was on the level of 39%. The enzymatic process proved to be an effective method for production of isoprenoid-phospholipid preparation enriched with citronellic acid. Proposed method is good alternative of obtaining this bioproduct in comparison with the chemical method because involves mild conditions, utilizes of natural substrates and commercially available regio-selective enzyme. Additional advantage includes the fact that this research obtained new biopreparation that contains unsaturated fatty acids in the *sn*-2 position, which possesses a beneficial healthy effect. This method is then promising in the context of the production of dietary enriched egg-yolk PC as food supplements containing terpenes for the prevention of cancer diseases and will be the subject of further studies on the enzymatic synthesis of isoprenoid-phospholipid.

## Figures and Tables

**Table 1:** Composition of fatty acids (wt %) of native egg-yolk PC and modified PC.

Fatty and CA Acids	Native PC	Modified PC
C10:1 (CA)	-	39
C16:0 (PA)	32	6
C16:1 (OPA)	1	1
C18:0 (SA)	14	2
C18:1 (OA)	27	27
C18:2 (LA)	21	21
C20:4 (AA)	5	4

wt = weight.

## References

[1] Harbone J.B., Harbone J.B., Tomas-Barberan F.A. (1991). Recent advances in the ecological chemistry of plant terpenoids. Proceedings of the Phytochemistry Society of Europe.

[2] Katsel P.L., Dmitrieva T.M., Valeyev R.B., Kozlov Y.P. (1992). Sex pheromones of male yellowfin Baikal sculpin (*Cottocomephorus grewingki*): Isolation and chemical studies. J. Chem. Ecol..

[3] Rao C.V., Newmark H.L., Reddy B.S. (2002). Chemopreventive effect of farnesol and lanosterol on colon carcinogenesis. Cancer Detect. Prev..

[4] Brehm-Strecher B.F., Johnson E.A. (2003). Sensitization of *Staphylococcus aureus* and *Escherichia coli* to antibiotics by the sesquiterpenoids nerolidol, farnesol, bisabolol, and apritone. Antimicrob. Agents Chemother..

[5] Peana A.T., D’Aquila P.S., Chessa M.L., Moretti M.D., Serra G., Pippia P. (2003). (−)-Linalool produces antinociception in two experimental models of pain. Eur. J. Pharmacol..

[6] Jansen D.J., Shenvi R.A. (2014). Synthesis of medicinally relevant terpenes: Reducing the cost and time of drug discovery. Future Med. Chem..

[7] Crowell P.L. (1999). Prevention and therapy of cancer by dietary monoterpenes. J. Nutr..

[8] Wang G., Tang W., Bidigare R.R., Zhang L., Demain A.L. (2005). Terpenoids as Therapeutic Drugs and Pharmaceutical Agents. Natural Products.

[9] Elson C.E., Yu S.G. (1994). The chemoprevention of cancer by mevalonate-derived constituents of fruits and vegetables. J. Nutr..

[10] Meadows S.M., Mulkerin D., Berlin J., Bailey H., Kolesar J., Warren D., Thomas J.P. (2002). Phase II trial of perillyl alcohol in patients with metastatic colorectal cancer. Int. J. Gastrointest. Cancer.

[11] Miquel A., Pradines G. (1996). Favre, Farnesol and geranylgeraniol induce actin cytoskeleton disorganization and apoptosis in A549 lung adenocarcinoma cells. Biochem. Biophys. Res. Commun..

[12] Ong T.P., Heidor R., De Conti A., Dagli M.L.Z., Moreno F.S. (2006). Farnesol and geraniol chemopreventive activities during the initial phases of hepatocarcinogenesis involve similar actions on cell proliferation and DNA damage, but distinct actions on apoptosis, plasma cholesterol and HMGCoA reductase. Carcinogenesis.

[13] Wiseman D.A., Werner S.R., Crowell P.L. (2007). Cell cycle arrest by the isoprenoids perillyl alcohol, geraniol and farnesol is mediated by p21Cip1 and p27Kip1 in human pancreatic adenocarcinoma cells. J. Pharmacol. Exp. Ther..

[14] Budrock G.A. (2010). Fenaroli’s Handbook of Flavor Ingredients.

[15] Lis-Balchin M., Roth G. (1999). Citronellic acid: A major component in two *Pelargonium* species (Geraniaceae). J. Essent. Oil Res..

[16] Wuryatmo E., Klieber A., Scott E.S. (2003). Inhibition of citrus postharvest pathogens by vapor of citral and related compounds in culture. J. Agric. Food Chem..

[17] Yamaguchi Y. (1997). Antimicrobial Compositions with Hinokitiol and Citronellic Acid. U.S. Patent.

[18] Phillips L.R., Malspeis L., Supko J.G. (1995). Pharmacokinetics of active drug metabolites after oral administration of perillyl alcohol, an investigational antineoplastic agent, to the dog. Drug Metab. Dispos..

[19] Hudes G.R., Szarka C.E., Adams A., Ranganathan S., McCauley R.A., Weiner L.M., Langer C.L., Litwin S., Yeslow G., Halberr T. (2000). Phase I pharmacokinetic trial of perillyl alcohol (NSC 641066) in patients with refractory solid malignancies. Clin. Cancer Res..

[20] Yamamoto Y., Hosokawa M., Kurihara H., Myashita K. (2008). Preparation of phosphatidylated terpenes via phospholipase D-mediated transphosphatidylation. J. Am. Oil Chem. Soc..

[21] Yamamoto Y., Hosokawa M., Kurihara H., Maoka T., Miyashita K. (2008). Synthesis of phosphatidylated-monoterpene alcohols catalyzed by phospholipase D and their antiproliferative effects on human cancer cells. Bioorg. Med. Chem. Lett..

[22] Gliszczyńska A., Niezgoda N., Gładkowski W., Czarnecka M., Świtalska M., Wietrzyk J. (2016). Synthesis and biological evaluation of novel phosphatidylcholine analogues containing monoterpene acids as potent antiproliferative agents. PLoS ONE.

[23] Gliszczyńska A., Niezgoda N., Gładkowski W., Świtalska M., Wietrzyk J. (2017). Isoprenoid-phospholipid conjugates as potential therapeutic agents: Synthesis, characterization and antiproliferative studies. PLoS ONE.

[24] Dahan A., Duvdevani R., Shapiro I., Elmann A., Finkelstein E., Hoffman A. (2008). The oral absorption of phospholipid prodrugs: In vivo and in vitro mechanistic investigation of trafficking of a lecithin-valproic acid conjugate following oral administration. J. Control. Release.

[25] Biodrowska K., Draus A.W., Gliszczyńska A., Gładkowski W., Leśniak A. (2012). Synthesis of isoprenoid phospholipids. Przem. Chem..

[26] Cui Y.M., Wei D.Z. (1997). Lipase-catalyzed esterification in organic solvent to resolve racemic naproxen. Biotechnol. Lett..

[27] Svensson I., Adlercreutz P., Mattiasson B. (1990). Interesterification of phosphatidylcholine with lipases in organic media. Appl. Microbiol. Biotechnol..

[28] Niezgoda N., Gliszczyńska A., Gładkowski W., Chojnacka A., Kiełbowicz G., Wawrzeńczyk C. (2016). Production of concentrates of CLA obtained from sunflower and safflower and their application to the lipase-catalyzed acidolysis of egg yolk phosphatidylcholine. Eur. J. Lipid Sci. Technol..

[29] Kaki S.S., Balakrishna M., Prasad R.B.N. (2014). Enzymatic synthesis and characterization of 1-lipoyl-2-palmitoyl phosphatidylcholine: A novel phospholipid containing lipoic acid. Eur. J. Lipid Sci. Technol..

[30] Kaki S.S., Adlercreutz P. (2011). Lipase-catalyzed synthesis and characterization of 1-butanoyl-2-palmitoyl phosphatidylcholine, a potential lipidic prodrug of butyric acid. Chem. Phys. Lipids.

[31] Adlecreutz P., Carrea G., Riva S. (2008). Fundamentals of Biocatalysis in Neat Organic Solvents. Organic Synthesis with Enzymes in Non-Agueous Media.

[32] He R., Du Y., Ling L., Ismail M., Hou Y., Yao C., Li X. (2017). Nanoformulation of dual bexarotene-tailed phospholipid conjugate with high drug loading. Eur. J. Pharm. Sci..

[33] Mutua L.N., Akoh C.C. (1993). Lipase-catalyzed modification of phospholipids: Incorporation of n-3 fatty acids into biosurfactants. J. Am. Oil Chem. Soc..

[34] Hossen M., Hernandez E. (2005). Enzyme-catalyzed synthesis of structured phospholipids with conjugated linoleic acid. Eur. J. Lipid Sci. Technol..

[35] Haresh T., Pandit A.B. (2010). Enzymatic acyl modification of phosphatidylcholine using immobilized lipase and phospholipase A_2_. Eur. J. Lipid Sci. Technol..

[36] Peng L., Xu X., Mu H., Hoy C.-E., Adler-Nissen J. (2002). Production of structured phospholipids by lipase-catalyzed acidolysis, optimization using response surface methodology. Enzyme Microbiol. Technol..

[37] Aura A.M., Forssell P., Mustranta A., Poutanen K. (1995). Transesterification of soy lecithin by lipase and phospholipase. J. Am. Oil Chem. Soc..

[38] Vikbjerg A.F., Mu H., Xu X. (2005). Parameters affecting incorporation and by-product formation during the production of structured phospholipids by lipase-catalyzed acidolysis in solvent-free system. J. Mol. Catal. B Enzym..

[39] Adlercreutz D., Budde H., Wehtje E. (2002). Synthesis of phosphatidylcholine with defined fatty acid in the *sn*-1 position by lipase-catalyzed esterification and transesterification reaction. Biotechnol. Bioeng..

